# T-Helper Type-2 Contact Hypersensitivity of Balb/c Mice Aggravated by Dibutyl Phthalate via Long-Term Dermal Exposure

**DOI:** 10.1371/journal.pone.0087887

**Published:** 2014-02-03

**Authors:** Jinquan Li, Li Li, Haoxiao Zuo, Chenjuan Ke, Biao Yan, Huaxiao Wen, Yinping Zhang, Xu Yang

**Affiliations:** 1 Section of Environmental Biomedicine, Hubei Key Laboratory of Genetic Regulation and Integrative Biology, College of Life Sciences, Central China Normal University, Wuhan, China; 2 Department of Building Science, Tsinghua University, Beijing, China; King's College London, United Kingdom

## Abstract

**Objective:**

During the last few decades, the prevalence of allergic skin diseases, asthma and rhinitis, has increased worldwide. Introduction of environmental chemicals with aggravation effects may play a part in this increase. The artificial chemical product dibutyl phthalate (DBP) is used in many products used in daily life. Dermal exposure to DBP is a common (but easily neglected) exposure pattern.

**Methodology/Principal Findings:**

In this study, we examined the aggravation effect of long-term dermal exposure to DBP in a T-helper type 2 (Th2) model of contact hypersensitivity (CHS) in mice, and sought the potential molecular mechanisms. Experimental tests were conducted after 40-day dermal exposure to saline or three concentrations of DBP and subsequent three times of sensitization with 0.5% fluorescein isothiocyanate (FITC) or vehicle. The results of immunological and inflammatory biomarkers (total-immunoglobulin (Ig)E and Th cytokines) as well as histopathological examination and measurement of ear swelling supported the notion that high doses of DBP may promote and aggravate atopic dermatitis. Increased expression of thymic stromal lymphopoietin (TSLP) in this mouse model suggested that TSLP might be one of the molecular mechanisms of the aggravation effect induced by DBP.

**Conclusions/Significance:**

Together, these results indicated that long-term dermal exposure to types of environmental toxins such as phthalates may endow an atopic predisposition in animals or humans. In addition, the high expression of TSLP in the mouse model demonstrated that TSLP might have an important role in the aggravation effect. This result could help to provide effective prevention strategies against atopic diseases such as atopic dermatitis (AD).

## Introduction

Phthalate esters are artificial chemical products. They are added to plastics to make them flexible and soft, to cosmetics as vehicles for fragrance, and to many other products, such as children's toys, building materials, and medical devices [1]–[3]. With the widespread use of phthalates in daily life, the potential consequences of human exposure to phthalates have raised concerns in the general population. Among all phthalate esters, dibutyl phthalate (DBP), which is one of the main sources of indoor semi-volatile organic compounds (SVOCs), has become the main type of plasticizer used in China [4]. Humans as well as animals can be exposed to these ubiquitous compounds through oral, dermal, inhalational, and iatrogenic exposures [5]. Dermal exposure is a common (but easily neglected) exposure pattern. Most chemicals are readily absorbed via the skin and can cause health effects and/or contribute to the dose absorbed by inhaling the chemical from the air. In many cases, skin is a more significant route of exposure than the lung [6]. Recent reports have revealed that phthalates are added to cosmetics (≤5%) and personal-care products, and that they raise the levels of exposure to urinary phthalate monoesters instantly if these products are used every day [7]. Hence, the toxicity of phthalates via dermal exposure needs more attention.

Atopic dermatitis (AD) is characterized by skin inflammation with dermal infiltration by T cells and eosinophils [8]. Over the past few decades, the prevalence of AD has been increasing in “developed” countries. Most patients with AD exhibit systemic T-helper type 2 (Th2)-dominated immune responses with elevated levels of immunoglobulin (Ig) E in serum [9]. Epidemiologic analysis shows that AD is often the initial step in the so-called “atopic march” [10]–[13], given that more than 50% of AD patients with moderate to severe AD develop asthma later in life, and the severity of AD influences the course of respiratory allergy. Therefore, if the severity of AD is aggravated, it will have a greater impact on human health. What’s more, both AD and asthma share an “atopy” phenotype that includes a Th2 inflammation with eosinophilia and hyper-IgE [11], [12]. Contact hypersensitivity (CHS) is a common type of AD. Various chemicals can induce CHS upon repeated exposure of the skin to them. This pathological reaction is mimicked readily in experimental animals by painting a particular chemical on the skin. Most types of CHS are mediated by Th1 cells. Interestingly, fluorescein isothiocyanate (FITC) induced CHS is dominantly mediated by Th2 rather than Th1 cells, although this model is an acute model [14]. Epidemiological studies have provided evidence for a possible relationship between phthalate exposure and the risk of allergy in children as well as in workers in the plastic industry [15]–[20]. Moreover, the increased prevalence of morbidity of AD corresponds with the increase in industrial pollution [21]. It is tempting to speculate that environmental chemicals, such as DBP, may increase the potency of allergens, and therefore play a crucial part in the enhancement of allergic diseases.

In models of CHS, dendritic cell (DC) activation by an immuno-active antigen is crucial for determining the pathway of the downstream responses mediated by T cells. One of the prime candidates for DC activation during an allergic inflammatory reaction is thymic stromal lymphopoietin (TSLP). Studies have determined that TSLP can “skew” the immune system toward Th2-dominance by activating DCs that promote differentiation of naïve T cells to Th2 cells by an OX40 ligand–OX40 interaction which then locally produces Th2 chemokines [22], [23]. Moreover, TSLP expression was significantly elevated in the epidermis of lesion skin from acute and chronic AD patients [24]. Interestingly, cytokines which are found at high levels in the skin of patients (tumor necrosis factor (TNF)-α, interleukin (IL)-4 and IL-13) can also synergize to induce TSLP expression, suggesting a feed-forward inflammatory cascade [25]. These findings suggest that TSLP plays an important part in the pathogenesis of AD. In addition, in previous studies, a mouse model to study human “atopic march” indicates that TSLP may represent an important factor in the link of atopic dermatitis to asthma [26]–[28].

Most studies illustrated the adjuvant effect of DBP using DBP as a solvent for FITC to induce CHS. However, no author has tried to explore the relationship between long-term exposure of DBP to the dermis and allergic diseases. Therefore, we studied in greater detail the suspected impact of DBP on AD using a long-term dermal exposure FITC-sensitized mice model. We aimed to address the aggravate effect of long-term DBP exposure on skin and allergic response to FITC related to AD and, by testing the expression of TSLP in skin, to seek the molecular mechanisms of DBP-induced aggravate effect in AD.

## Materials and Methods

All protocols used in these studies were approved by the Office of Scientific Research Management of Central China Normal University (Beijing, China; 8 November 2011; CCNU-SKY-2011-008).

### Experimental animals

Male Balb/c mice (5–6 weeks; 22±1.5 g) were purchased from the Hubei Province Experimental Animal Center (Wuhan, China). They were housed in pathogen-free cages maintained at 24–26°C with 55–75% humidity and a 12-h light–dark cycle. They were fed a commercial diet (Hubei Province Experimental Animal Center) and given water *ad libitum*. Mice were quarantined for ≥7 days before study initiation. Eight mice in each group were utilized so as to minimize the number of experimental animals needed while ensuring the validity of statistical power.

### Main reagents and kits

DBP (>99%), FITC, formalin solution (10%) and pentobarbital sodium were purchased from Sigma–Aldrich (St. Louis, MO, USA). Tween-80 was obtained from Amresco (Solon, OH, USA). All other chemicals were of analytical grade. Mouse enzyme-linked immunosorbent assay (ELISA) kits for total IgE was purchased from Biolegend (San Diego, CA, USA). Mouse ELISA kits for IL-4, interferon (IFN)-γ, IL-17A, TSLP and TNF-α were from eBioscience (San Diego, CA, USA). Mouse anti-IL-5-antibody, mouse anti-IL-13-antibody, and mouse anti-eosinophil cationic protein (ECP)-antibody, goat-anti-rabbit lgG-antibody, a rabbit lgG peroxidase conjugated streptavidin-biotin complex (SABC-POD) kit and a diaminobenzidine (DAB) kit were obtained from Boster Bio-engineering (Wuhan, China).

### Exposure and immunization protocol

**Figure 1:**
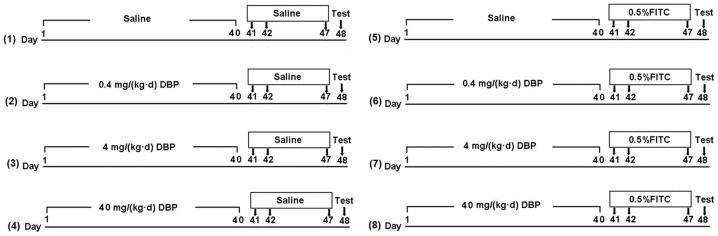
Exposure and immunization schedule. To make long-term dermal exposure, male Balb/c mice were smeared with 0.1 mL saline or DBP (0.4, 4.0 and 4.0 mg/(kg•d)) from day 1 to 40 (40 times) on their shaven backs, and then sensitized with 120 µL of saline or 0.5% FITC (in 1∶1 acetone/DBP) on days 41 and 42, on their shaven backs. On day 47, baseline ear thickness was measured with calipers followed by challenge with 20 µL of saline or 0.5% FITC on the right ear, and saline or vehicle (1∶1 DBP/acetone) on the left ear. n = 8 mice in each group. (1) Saline, (2) DBP 0.4, (3) DBP 4, (4) DBP 40, (5) FITC, (6) FITC+DBP 0.4, (7) FITC+DBP 4, (8) FITC+DBP 40.

The long-term treatments with DBP were done by the application of aqueous suspension, in which DBP was diluted in sterile saline from stock solution. The stock solution was prepared by dissolving DBP in Tween 80 (1∶1 ratio). Skin sensitization and application to ears with 0.5% FITC were performed in acetone-based solvent, in which acetone and DBP (1∶1 ratio) were always present. Male Balb/c mice were divided randomly into eight groups of 8 mice: (i) Saline skin exposure combined with saline-treated group (Saline); (ii) 0.4 mg/(kg•d) DBP skin exposure combined with saline-treated group (DBP 0.4); (iii) 4.0 mg/(kg•d) DBP skin exposure combined with saline-treated group (DBP 4); (iv) 40.0 mg/(kg•d) DBP skin exposure combined with saline-treated group (DBP 40); (v) Saline skin exposure combined with 0.5% FITC sensitized group (FITC); (vi) 0.4 mg/(kg•d) DBP skin exposure combined with 0.5% FITC sensitized group (FITC+DBP 0.4); (vii) 4.0 mg/(kg•d) DBP skin exposure combined with 0.5% FITC sensitized group (FITC+DBP 4.0) (viii) 40.0 mg/(kg•d) DBP skin exposure combined with 0.5% FITC sensitized group (FITC+DBP 40). The detailed protocols are shown in Figure 1.

### Serum sample preparation and quantitative analyses of IgE

On day 48, 24 h after challenge, mice were anesthetized with pentobarbital sodium (100 mg/kg, i.p.). Serum samples were collected from heart blood by centrifugation (3000 rpm, 15 min, 24°C) and stored at −70°C until analyses. Serum levels of total IgE (T-IgE) were measured using an ELISA kit according to manufacturer protocols. The sensitivity of the kit was 15 pg/mL. Concentrations were determined in duplicate for each sample.

### Measurement of ear swelling and difference in bilateral ear weight

After collecting serum, mice were killed by cervical dislocation. Ear swelling was measured using a vernier caliper. Ear edema was expressed as (R±L) ± (R0±L0), where R0 and L0 represent the thickness of the right and left ear, respectively, at the beginning of sensitization (40 d), and R and L stand for the thickness values obtained on day 48. Then, ears were cut and punched along the edge of the middle ear by a corneal trephine of diameter 8 mm. The weight was measured immediately, and the difference in bilateral ear weight calculated.

### Histological examination

After measurement of ear swelling and ear weight, the right ears of mice were collected and fixed overnight in 10% formalin at room temperature. They were then sectioned, and stained with hematoxylin and eosin or toluidine blue as reported previously [29]. Tissue sections were observed using a DM 4000B Microscope (Leica, Berlin, Germany). The numbers of inflammatory cells and degranulated mast cells in each sample were counted using Image-Pro Plus software (Image-Pro Plus 6.0, Media Cybernetics). A non-stained region was selected and set as the background. All tissue sections were examined qualitatively by two experienced pathologists in a blinded fashion.

### Tissue sample preparation and ELISA

The right ears of mice were removed 24 h after the final challenge. They were homogenized using a glass homogenizer on ice using 10 mL/g of ice-cold phosphate-buffered saline (PBS; pH 7.5) to make a 10% tissue homogenate. Afterwards, the homogenate was used to test the levels of IL-4, IFN-γ, TSLP, IL-17A and TNF-α according to manufacturer instructions using commercial ELISA kits. The sensitivities of the kits were 4 pg/mL for IL-4, 15 pg/mL for IFN-γ, 16 pg/mL for TSLP, 4 pg/mL for IL-17A and 8 pg/mL for TNF-α. Concentrations were determined in duplicate for each sample.

### Immunohistochemical assay

Sections of ear tissues were quenched of endogenous peroxides with 3% H_2_O_2_. They were boiled in sodium citrate (0.01 mol/L, pH 6.0) for antigen retrieval to unmask antigen epitopes, permeabilized with 0.2% Triton X-100 for 10 min, and blocked with 5% BSA in PBS for 30 min at room temperature. Sections were incubated with diluted primary antibodies (mouse anti-IL-5antibody, mouse anti-IL-13 antibody or mouse anti-ECP antibody, 1∶200 dilution) overnight at 4°C. Slides were washed with PBS, incubated with secondary antibody (anti-rabbit IgG, 1∶200 dilutions) for 30 min at 37°C and detected with a rabbit IgG peroxidase conjugated streptavidin-biotin complex (SABC-POD) kit, followed by incubation with a diaminobenzidine (DAB) kit. Immunostained sections were viewed under a DM 4000B Microscope (Leica). The intensity of protein IL-5, IL-13 or ECP staining was determined as average optical density using Image-Pro Plus 6.0 software. A non-stained region was selected and set as the background. Two experienced pathologists in a blinded fashion examined all tissue sections qualitatively.

### Statistical analyses

Data are the mean ± standard error of the mean. Statistical graphs were generated using Origin 8.0 Software (OriginLab, Berkeley, CA, USA). All data were analyzed by a Oneway ANOVA followed by Tukey test. A *p*-value of *p*≤0.05 was considered as significant. Data analyses were carried out using SPSS ver13 (SPSS, Chicago, IL, USA).

## Results

### Effect of dermal exposure DBP on serum levels of IgE

**Figure 2:**
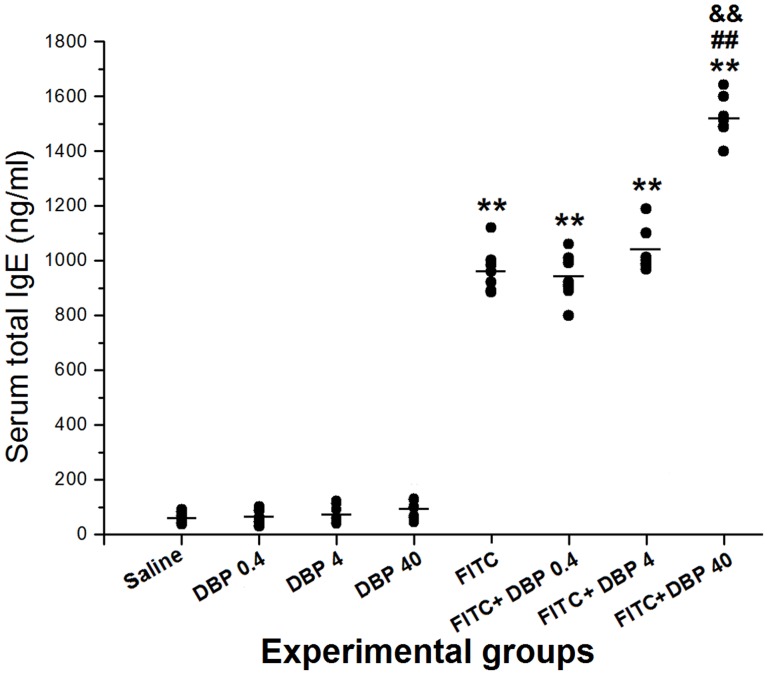
Serum IgE levels. **: *p*<0.01, compared with the saline group; ##: *p*<0.01, compared with the FITC group; &&: *p*<0.01 compared with the FITC+DBP 4 group.

To evaluate the aggravation effect of skin exposure to DBP for Ig production, we measured T-IgE in serum 24 h after the final challenge. Figure 2 shows the measurement data of serum T-IgE after DBP treatment in the absence or presence of FITC sensitization. Four important findings were revealed: (i) exposure to only DBP (DBP 0.4, DBP 4 and DBP 40) did not cause changes in serum T-IgE; (ii) the T-IgE levels of all FITC-immunized groups (FITC, FITC+DBP 0.4, FITC+DBP 4, FITC+DBP 40) were increased significantly in relation to those of the saline exposure group (*p*<0.01); (iii) compared with the FITC group, FITC+DBP 40 (*p*<0.01) enhanced the level of T-IgE (which suggested that a aggravation effect occurred at high levels of DBP combined with allergen); (iv) the T-IgE levels of the FITC+DBP 40 group were increased significantly compared with those of the FITC+BDP 4 group (*p*<0.01).

### Effect of dermal exposure to DBP on skin lesions

**Figure 3:**
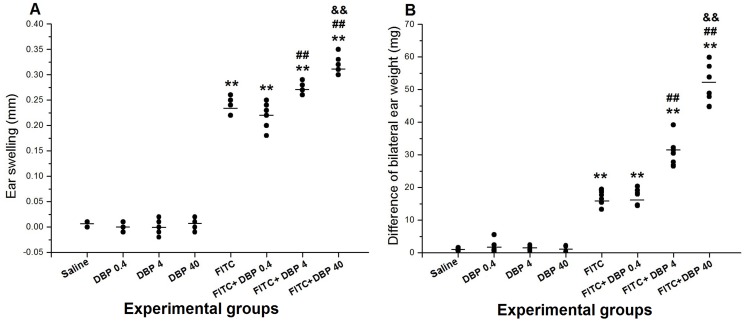
Effects of skin exposure DBP on skin lesions induced by FITC. (A) Measurement results of ear swelling. (B) Measurement results of differences in bilateral ear weight. **: *p*<0.01, compared with the saline group; ##: *p*<0.01, compared with the FITC group; &&: *p*<0.01 compared with the FITC+DBP 4 group.

To evaluate the effects of DBP on skin in the presence or absence of FITC, we examined ear swelling and differences in bilateral ear weight 24 h after the final challenge. Figure 3 shows the measurement results of ear swelling and differences in bilateral ear weight. The results showed two features: (i) compared with the saline group, FITC groups (FITC, FITC+DBP 0.4, FITC+DBP 4, FITC+DBP 40) caused significant changes with regard to ear swelling and differences in bilateral ear weight; (ii) compared with the 0.5% FITC-sensitized group, FITC+DBP 4 and FITC+DBP 40 groups enhanced ear swelling and differences in bilateral ear weight (*p*<0.01) and, with an increasing concentration of DBP, the aggravation effect was stronger.

### Effect of dermal exposure to DBP on histological examination

**Figure 4:**
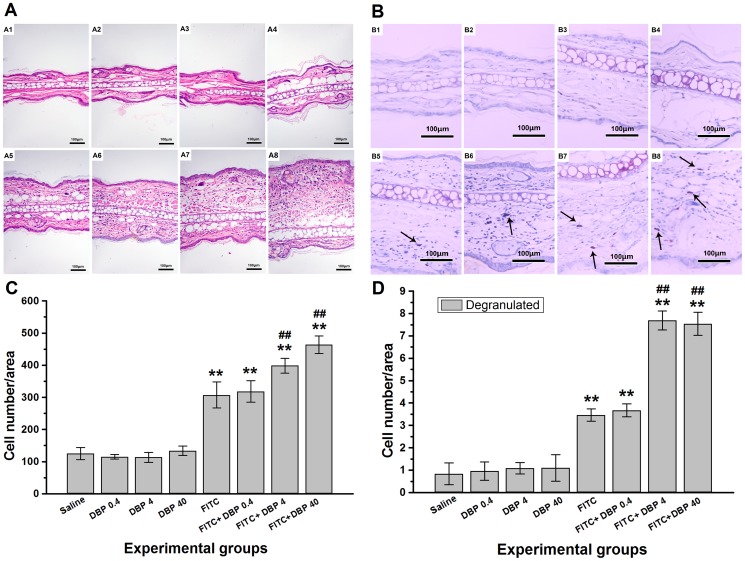
Effects of skin exposure to DBP on histological changes in the ear. (A) Stained with hematoxylin & eosin (H&E). (B) Stained with toluidine blue, degranulation mast cells were stained purple color (black arrow). (C) Number of inflammatory cells infiltrating. (D) Number of mast cells degranulating. In (A) and (B), A1–A8 or B1–B8 represent different exposure groups (saline, DBP 0.4, DBP 4, DBP 40, FITC, FITC+DBP 0.4, FITC+DBP 4, FITC+DBP 40). **: *p*<0.01, compared with the saline group; ##: *p*<0.01, compared with the FITC group.

To evaluate histological changes, we carried out hematoxylin and eosin (H&E) (Fig. 4A) and toluidine blue staining (Fig. 4B) 24 h after the final challenge. Exposure to saline (Fig. 4 A1, Fig. 4 B1) or DBP (Fig. 4 A2–A4, Fig. 4 B2–B4) did not lead to significant pathologic alterations. The FITC group caused inflammatory cells infiltration into the skin compared with the saline group (Fig. 4 A5, C: *p*<0.01). Furthermore, combined administration of FITC+DBP (FITC+DBP 4, FITC+DBP 40) increased the number of inflammatory cells infiltrating compared with FITC group (Fig. 4 A5–A8, C: *p*<0.01). A similar trend was observed regarding the severity of degranulation of mast cells (Fig. 4B, D).

### Effect of dermal exposure to DBP on eosinophilic infiltration

**Figure 5:**
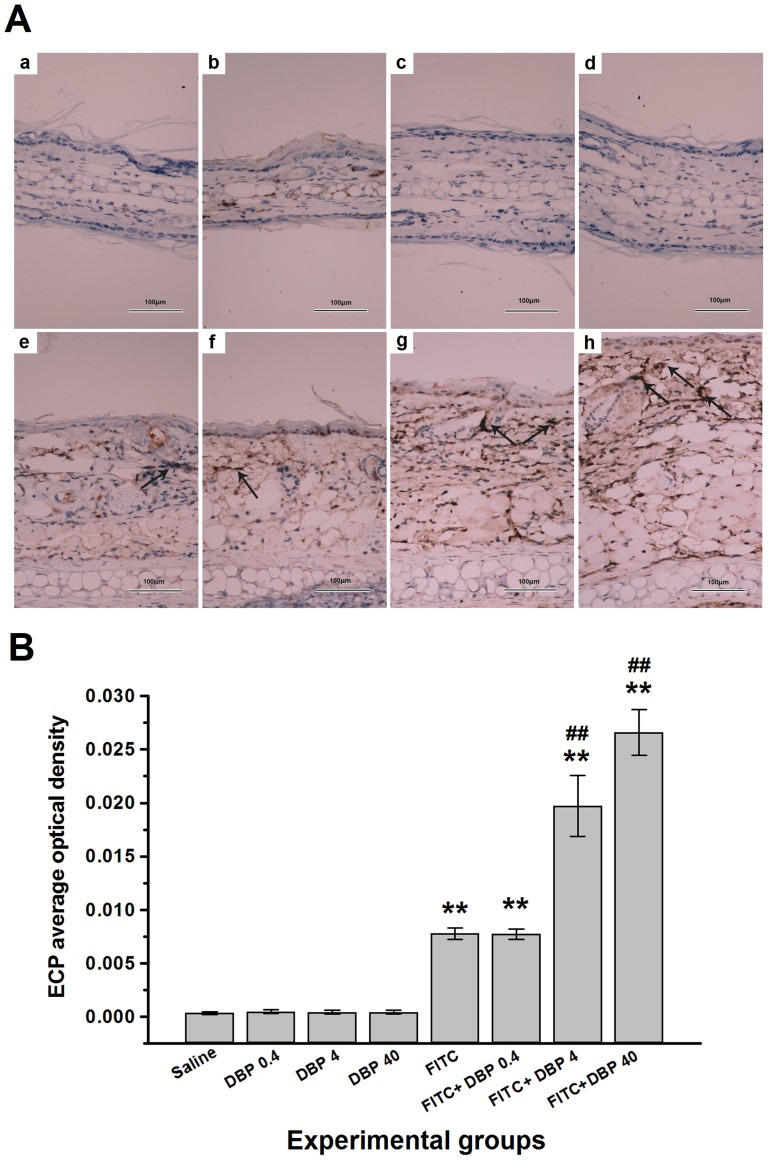
Immunohistochemical analyses. Representative images of the expression of (A) eosinophil cationic protein (ECP) as determined by immunohistochemical staining (brown color stain) (black arrow). Panel: (a) Saline, (b) DBP 0.4, (c) DBP 4, (d) DBP 40, (e) FITC, (f) FITC+DBP 0.4, (g) FITC+DBP 4, (h) FITC+DBP 40. Analyses of (B) ECP expression level according to average optical density. Animal groups (in all panels): n = 4 mice per group. **: *p*<0.01, compared with saline group; ##: *p*<0.01, compared with FITC group.

Eosinophils play a major role in AD and become active by releasing their toxic eosinophilic granules, which constitute a major portion of their cellular protein content [30]. Eosinophil cationic protein (ECP) is exclusively secreted only by the eosinophilic leukocyte [31]. Therefore, we use immunohistochemical experimentation to test the concentration of ECP in order to reflect the eosinophilic infiltration. The average optical density was measured. We noted in Fig. 5 that the FITC group presented an increase in the content of ECP compared with the saline group (*p*<0.01). Combined administration of FITC+DBP (FITC+DBP 4, FITC+DBP 40) exposure significantly enhanced the levels of ECP compared with FITC group (*p*<0.01).

### Effect of dermal exposure DBP on levels of Th cytokines in the skin

**Figure 6:**
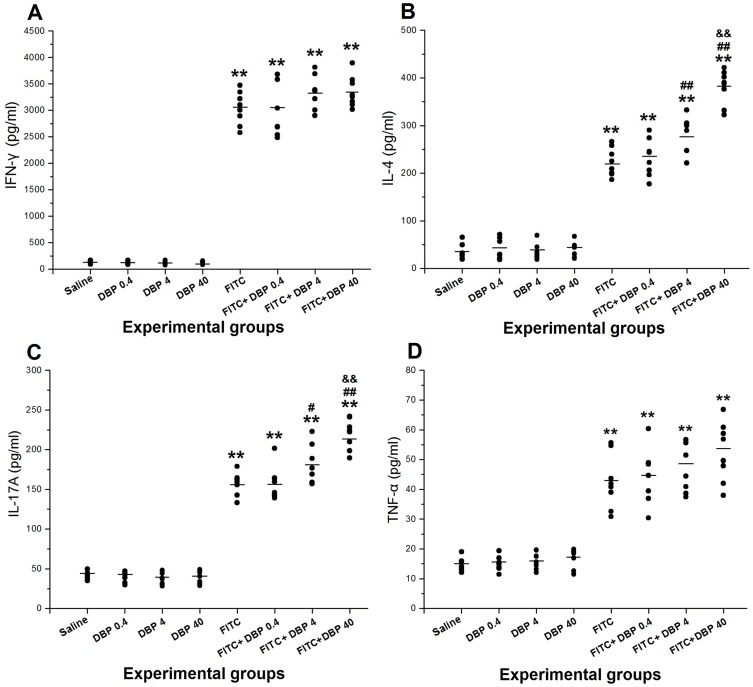
Effects of skin exposure to DBP on the protein expression of Th Cytokines in the ear. (A) Levels of IFN-γ. (B) Levels of IL-4. (C) Levels of IL-17A. (D) Levels of TNF-α. **: *p*<0.01, compared with the saline group; ##: *p*<0.01, compared with the FITC group; &&: *p*<0.01 compared with the FITC+DBP 4 group.

**Figure 7:**
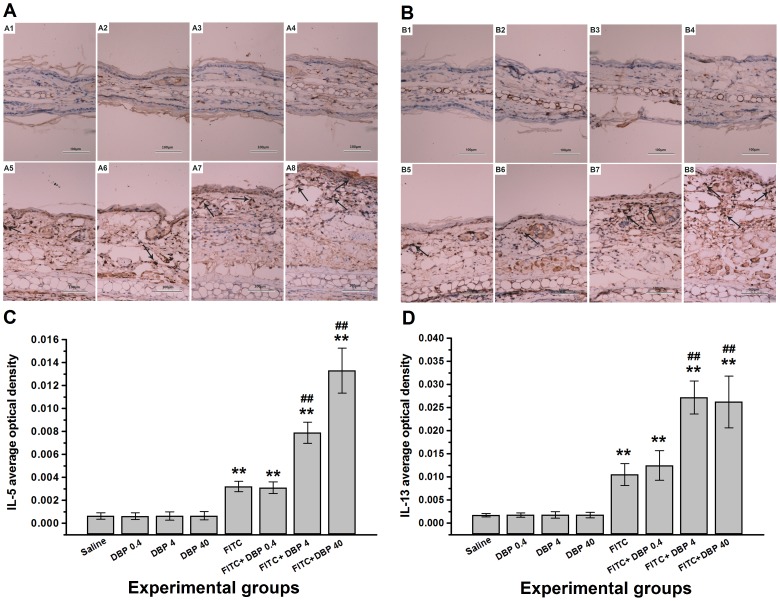
Immunohistochemical analyses. Representative images of the expression of (A) IL-5 and (B) IL-13 as determined by immunohistochemical staining (brown color stain) (black arrow). Panel: (A1, B1) Saline, (A2, B2) DBP 0.4, (A3, B3) DBP 4, (A4, B4) DBP 40, (A5, B5) FITC, (A6, B6) FITC+DBP 0.4, (A7, B7) FITC+DBP 4, (A8, B8) FITC+DBP 40. Analyses of (C) IL-5 and (D) IL-13 expression levels according to average optical density. Animal groups (in all panels): n = 4 mice per group. **: *p*<0.01, compared with saline group; ##: *p*<0.01, compared with FITC group.

The levels of the Th1 cytokine IFN-γ, the Th2 cytokine IL-4 and the Th17 cytokine IL-17A were assessed in ear tissue samples (Fig. 6A, B and C). To evaluate the effects of skin exposure to DBP on TNF-α release in the presence or absence of allergen, we measured TNF-α level in ear tissue 24 h after the final challenge (Fig. 6D). Immunohistochemical analyses were conducted to detect the expression of the other two Th2 cytokines IL-5 and IL-13 in ear tissue (Fig. 7). Exposure to only DBP (DBP 0.4, DBP 4, DBP 40) did not cause changes in the levels of IFN-γ, IL-4, IL-5, IL-13, IL-17A and TNF-α compared with the saline control group. The protein levels of IFN-γ, IL-4, IL-5, IL-13, IL-17A and TNF-α in the FITC-immunized groups were significantly greater than those in the saline group (*p*<0.01). Administration of 4.0 and 40.0 mg/(kg·d) DBP combined with FITC led to a further significant increase in the levels of IL-4, IL-5, IL-13 and IL-17A compared with FITC group. However, skin exposure to DBP did not elevate the expression of IFN-γ and TNF-α in the presence of FITC compared with the FITC group.

### Effect of dermal exposure to DBP on TSLP expression in the skin

**Figure 8:**
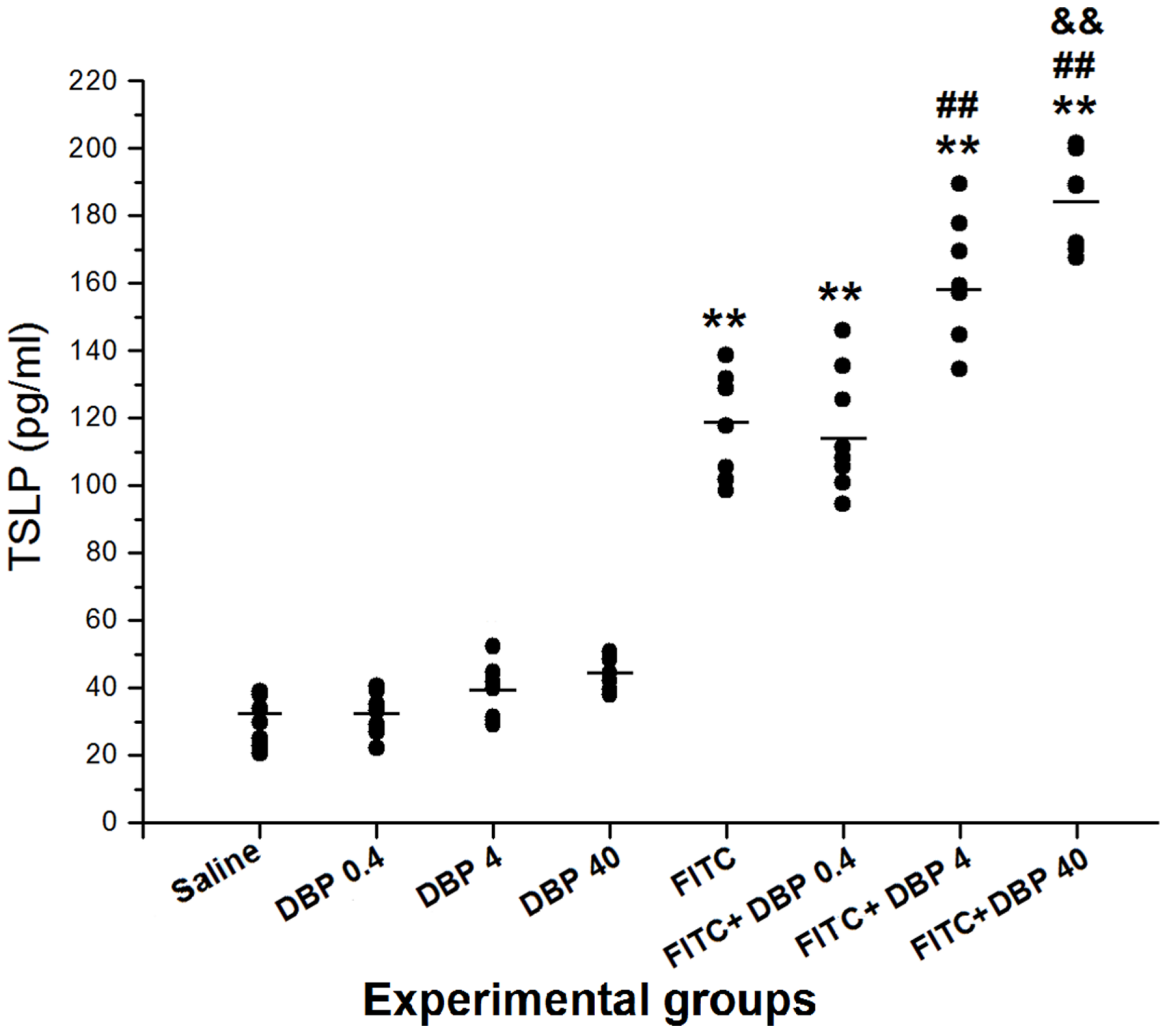
Effect of skin exposure to DBP on TSLP expression in the ear. **: *p*<0.01, compared with the saline group; ##: *p*<0.01, compared with the FITC group; &&: *p*<0.01 compared with the FITC+DBP 4 group.

Given that TSLP is an important cytokine produced by epithelial cells and has been linked to AD, we measured TSLP expression in the skin after sensitization with FITC (Fig. 8). The DBP 0.4, DBP 4 and DBP 40 groups did not cause changes in TSLP levels compared with the saline group. The TSLP levels of all FITC-immunized groups were increased significantly compared with the saline group (*p*<0.01). Co-exposure with FITC, 4.0 and 40.0 mg/(kg·d) DBP exposure groups showed a strong aggravation effect on the results of TSLP (*p*<0.01), especially at the highest dose of 40.0 mg/(kg·d).

## Discussion

**Figure 9:**
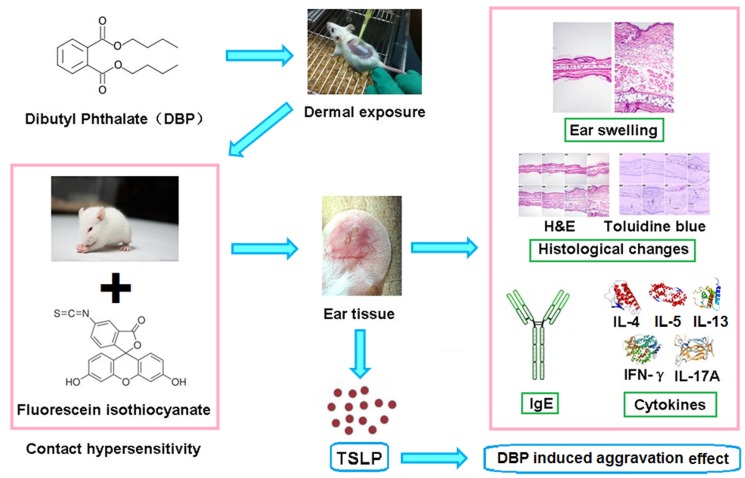
Graphical summary of the study.

The present study showed that dermal exposure of 4.0 and 40.0 mg/(kg·d) DBP could aggravate AD-like skin lesions related to FITC-induced CHS in mice. This deterioration was concomitant with increased skin levels of Th2 cytokines such as IL-4, IL-5 and IL-13; Th17 cytokines such as IL-17A, respectively. Also, 4.0 and 40.0 mg/(kg·d) DBP exhibited aggravation effect for the production of IgE in the presence of allergen. Furthermore, 4.0 and 40.0 mg/(kg·d) DBP potentiated the accumulation of eosinophils and the degranulation of mast cells in the skin in the presence of allergen. Finally, DBP could promote the expression of TSLP in the skin (Fig. 9).

With the widespread use of DBP, the potential toxicity is getting increasing attention. Several studies have demonstrated that ingestion exposure to DBP can affect male fertility, cause testicular atrophy in young adults, and produce teratogenicity and embryomortality in rodents [3], [32]–[35]. The effects of DBP on the skin (another major route of exposure other than the ingestion) remains incompletely understood, although they are widely used in various cosmetic products. The Taiwan Food and Drug Administration (TFDA) announced on 31 July 2011 the tolerable daily intake (TDI) for DBP to be 0.01 mg/(kg·d). According to this limit and the different drug use between humans and mice, the mice in the present study were treated every day with dermal exposure of 0, 0.4, 4, and 40 mg/(kg·d) DBP solution, respectively. Forty daily dermal exposures to DBP rather than a few immediately-high-dose exposures were administrated before allergen challenge to simulate the real environmental exposure.

FITC-mediated CHS in mice has been characterized as an animal model of allergic contact dermatitis mediated by a Th2-dominant immune system: high amounts of IL-4 were expressed at the protein level in the inflamed ear, and plasma IgE levels were increased [36], [37]. DBP components have been reported to act as indispensable adjuvants for FITC-induced CHS [14]. However, it is unclear if long-term dermal exposure to DBP could exacerbates this Th2 contact hypersensitivity. In the present study, the aggravation effect of DBP was evaluated through two groups of important biomarkers: “immune response” and “AD pathology”. The former include IgE, IL-4, IL-5, IL-13, IFN-γ, IL-17A and TNF-α in the skin, and the latter include histological and physiological changes in the skin. We observed that, in the absence of FITC, exposure to DBP alone did not cause obvious histological and physiological changes in mice. However, after 40 days dermal exposures, compared with the FITC group, 4.0 and 40.0 mg/(kg·d) DBP+FITC exposure groups could aggravate the levels of IgE, IL-4, IL-5, IL-13 and IL-17A in the skin, histological changes such as eosinophil infiltration and degranulation of mast cells in the skin, and physiological changes such as ear swelling. These phenomena suggested that long-term exposure to DBP combined with FITC-induced CHS could exacerbate AD-like symptoms.

Studies have demonstrated the role of TSLP in the regulation of Th2 responses in allergic disease from cell different lines. First, TSLP-treated DCs can prime helper T cells to present a Th2 phenotype in humans and mice, and the expression of TSLP is markedly elevated in the lesional skin of individuals with AD [23], [24]. Likewise, in mice, TSLP expression in the skin is increased in a model of FITC-induced allergic contact dermatitis, whereas TSLPR^−/−^ mice are resistant to disease development [38]. Moreover, over-expression of TSLP in the skin leads to the development of spontaneous disease that closely resembles human AD [39]. Recently, Boehme *et al*. showed that treatment with FITC in acetone and DBP, or acetone and DBP alone, resulted in increased levels of TSLP protein in the skin [40]. We have now tested the concentration of TSLP in skin in an antigen-driven model of skin inflammation in mice, CHS, which combined with 40-day dermal exposure of DBP. In the present study, after 40 days of dermal exposure, the expression of TSLP in 4.0 and 40.0 mg/(kg·d) DBP+FITC exposure groups was increased significantly compared with that in the FITC group. This result coincided with expression of those biomarkers used to evaluate the aggravation effect of DBP. These evidences suggested that the aggravation effect of DBP during the sensitization phase of CHS may (at least in part) be mediated by TSLP.

TSLP has been shown to inhibit Th1 and Th17 responses in the intestine [25], [41]. However, we did not observe decreased levels of IFN-γ or IL-17A in the inflamed tissue of FITC-exposure groups. Even though, with the increased concentration of DBP, the level of IFN-γ was not reduced in FITC-sensitization groups, the level of IL-4, IL-5 and IL-13 were increased significantly. This finding suggested that TSLP played an important part in skewing the immune system toward a Th2 response. The increased level of IL-17A also suggested that TSLP might take part in activation of the Th17 response. Nowadays there is sufficient experimental evidence to classify AD amongst other inflammatory skin disorders as IL-17 associated diseases. IL-17 is regarded as a signature cytokine for Th17 cells and is involved in inflammatory responses as observed in allergic reactions such as CHS [42]. In several human studies, T-cell-clones could be isolated from eczema biopsies, and high IL-17 levels were observed after challenge with allergen [43]. The indications for a participation of Th17 in the development of AD are supported by data from IL-17 deficient mice with reduced CHS reactions that could be restored after transplantation of wild type CD4+ T cells [44]. The results from Th17 cell research allow today identification of different skin diseases by a specific profile of signature cytokines from Th cells that can be used as a future diagnostic tool.

The present study is the first report to show that long-term dermal exposure to DBP can aggravate allergic contact dermatitis in mice. These evidences provide new explanations of the acquirement of an atopic predisposition and the increased prevalence of allergic disease in society. That is, that long-term dermal exposure to types of environmental toxins such as phthalates may endow an atopic predisposition in animals or humans. In addition, the high expression of TSLP in the mouse model demonstrated that TSLP might have an important role in the aggravation effect. This result could help to provide effective prevention strategies against atopic diseases such as AD. Meanwhile, transient receptor potential ankyrin 1 (TRPA1) activation may be another useful marker to find chemicals that facilitate sensitization in combination with other immunogenic haptens [45]. Therefore, more studies are needed for understanding the molecular mechanism on the aggravation effect of DBP in order to effectively prevent related health problems in the future.
